# Therapeutic and Prophylactic Effects of Amphotericin B Liposomes on Chronic Social Defeat Stress-Induced Behavioral Abnormalities in Mice

**DOI:** 10.3389/fphar.2022.918177

**Published:** 2022-07-15

**Authors:** Jiashu Lu, Chao Huang, Qun Lu, Xu Lu

**Affiliations:** ^1^ Department of Pharmacy, The People’s Hospital of Taizhou, The Fifth Affiliated Hospital of Nantong University, Taizhou, China; ^2^ Department of Pharmacology, School of Pharmacy, Nantong University, Nantong, China; ^3^ Department of Pharmacy, Nantong Third Hospital Affiliated to Nantong University, Nantong, China

**Keywords:** amphotericin B liposome, therapeutic effect, prophylactic effect, innate immune response, behavioral abnormalities

## Abstract

Recently, innate immune system stimulants, such as lipopolysaccharide (LPS) and macrophage-colony stimulating factor (M-CSF), were reported to prevent and reverse chronic stress-induced behavioral abnormalities, suggesting that innate immune stimulation could be a potential strategy for the treatment and prevention of mental disorders. Amphotericin B liposome is a clinically available antifungal medication that can stimulate macrophages and microglia. We hypothesize that amphotericin B liposome may be used to prevent and reverse behavioral abnormalities triggered by chronic stress. As expected, our results showed that a single injection of amphotericin B liposome (1 mg/kg) immediately after stress cessation reversed the decrease in time spent in the interaction zone in the social interaction test (SIT) and the increase in immobility time in the tail suspension test (TST) and forced swimming test (FST) in mice caused by chronic social defeat stress (CSDS). In addition, a single injection of amphotericin B liposomes (1 mg/kg) 1 day before stress exposure was found to prevent the CSDS-induced decrease in time spent in the interaction zone in the SIT and the increase in immobility time in the TST and FST in mice. Pretreatment with minocycline to inhibit the innate immune response was able to abolish the reversal effect of post-stress injection of amphotericin B liposomes on CSDS-induced behavioral abnormalities and the prophylactic effect of pre-stress injection of amphotericin B liposomes on CSDS-induced behavioral abnormalities. These results demonstrate that amphotericin B liposomes have both therapeutic and prophylactic effects on chronic stress-induced behavioral abnormalities in mice by mobilizing the innate immune response.

## Introduction

Chronic stress is a common phenomenon in modern society. It can cause the emergence of abnormal behaviors that cause severe mental disorders (Brenhouse et al., 2019). The currently used medications to treat behavioral abnormalities in mental disorders such as depression cannot meet the therapeutic requirements (Köhler et al., 2016; Vahid-Ansari and Albert, 2021), because only a proportion of patients treated with behavior-improving medications show good therapeutic effect (Schwartz et al., 2016), and these clinically used medications can induce many adverse effects, such as inducing suicide thoughts and insomnia (Edinoff et al., 2021; Hengartner et al., 2021). Therefore, the development of new strategies that can prevent or reverse the harmful influences of chronic stress on mood and behavior is of great importance for improving mental health and reducing the morbidity of mental disorders.

The innate immune system, composed mainly of macrophages and microglia, is an endogenous defense system that can protect the body from environmental stresses (Devanney et al., 2020; Gopinath et al., 2020). Overactivation of the innate immune response plays a key role in triggering behavioral abnormalities in rodents (Dey and Hankey Giblin, 2018; Li et al., 2022). Increased serum levels of pro-inflammatory cytokines derived from the over-activated innate immune cells have been repeatedly observed in patients with mental disorders such as depression (Yao et al., 2020; Ganança et al., 2021). Therefore, suppression of neuroinflammation has long been considered a potential strategy to treat mental disorders (Tateishi et al., 2022; Tayab et al., 2022). However, this view has been challenged by some other evidence. For example, researchers have found that some patients with depression have normal or even reduced pro-inflammatory markers in serum (Whooley et al., 2007; Almeida et al., 2009; Dowlati et al., 2010; Lehto et al., 2010), and some classical anti-inflammatory drugs such as cyclo-oxygenase-1 (COX-1) inhibitors and non-steroidal anti-inflammatory drugs (NSAIDs) have been found to have no antidepressant efficacy and even cause depressive symptoms (Browning, 1996; Choi et al., 2009). We and others had reported that chronic stress can induce a remarkable decrease in the number of microglia in the dentate gyrus of the hippocampus and that reversal of this decrease by innate immune system stimulants can reverse the behavioral abnormalities induced by chronic stress in mice (Kreisel et al., 2014; Tong et al., 2017; Cai et al., 2020). Moreover, moderate activation of the innate immune response may not only produce a toxic effect but also neuroprotective effects in various models of central nervous system disorders such as cerebral ischemia (Stevens et al., 2011; Vartanian et al., 2011) and traumatic brain or spinal cord injury (Longhi et al., 2011; Li et al., 2013). In previous studies, we had reported that a single injection of a low dose of lipopolysaccharide (LPS) or macrophage-colony stimulating factor (M-CSF) before stress exposure prevented the development of abnormal behaviors in chronically stressed mice by mobilizing the innate immune response (Gu et al., 2021; Ji et al., 2021). Thus, stimulating the innate immune system may be a novel strategy to prevent the development of behavioral abnormalities or ameliorate existing behavioral abnormalities under chronic stress.

Amphotericin B is a clinically available antifungal medication. It has been reported to promote the production of pro-inflammatory cytokines in cultured and bone marrow-derived microglia and macrophages (Motoyoshi et al., 2008; Döring et al., 2015) and, by activating innate immune cells, to exert efficient therapeutic effects in a number of models of neurological disease, including ameliorating lysolecithin-induced demyelination of the spinal cord (Döring et al., 2015) and prolonging the life-span of mice harboring patient-derived brain tumor initiating cells (Sarkar et al., 2014). Although amphotericin B can activate the innate immune cells, its administration cannot reverse behavioral abnormalities induced by chronic stress (Gao et al., 2019). Amphotericin B liposome is a liposomal form of amphotericin B. Unlike amphotericin B, amphotericin B liposome is more lipophilic and therefore has a stronger ability to penetrate the blood-brain barrier, which would result in a higher concentration of amphotericin B liposome in brain tissue (Zhang et al., 2003; O'Connor et al., 2013). Given its potential effect on stimulating innate immune cells, we speculate that amphotericin B liposome may be able to prevent and reverse behavioral abnormalities triggered by chronic stress. As expected, our results show that a single injection of amphotericin B liposome has both a therapeutic and a prophylactic effect on the behavioral abnormalities induced by chronic social defeat stress (CSDS) in mice, in a manner that is dependent on the stimulation of the innate immune system. These findings may help us to develop new strategies for the prevention and treatment of mental disorders.

## Materials and Methods

### Experimental Animals

C57BL6/J mice, 6-8-weeks-males, were purchased from the Model Animal Research Center in Nanjing University (Nanjing, China). Mice were kept in groups under standard conditions (12-h light/dark cycle; lights on from 07:00 to 19:00; 23 ± 1°C ambient temperature; 55% ± 10% relative humidity) with free access to food and water. Animal experiments were conducted in accordance with the internationally accepted guidelines for the use of animals in toxicology as adopted by the Society of Toxicology in 1999 and approved by the Animal Ethics Committee of Nantong University (Permit Number: 2110836). Researchers were blind to group assignment during data analysis.

## Materials

The minocycline is a product of Sigma (Saint Louis, MO, United States ), and the amphotericin B liposome was purchased from Shanghai New Pioneer Pharmaceutical Co., Ltd., (Shanghai, China). Both amphotericin B liposome and minocycline were dissolved in saline, and the saline without amphotericin B liposome was used as vehicle.

### Construction of the CSDS Model

The CSDS model was constructed following one of our previous studies (Gu et al., 2021). Briefly, 1 week before social stress stimulation, aggressive CD1 mice were selected based on the following criteria for 3 days: the latency of the first attack of CD1 was <90 s but longer than 5 s; the CD1 mouse attacked on at least 2 consecutive days during the 3-days selection process. During the process of social defeat stress, each C57BL6/J mouse (subject) was exposed to a novel aggressive CD1 mouse for up to 10 min (10 days) each day. Thereafter, subjects were separated from the CD1 aggressor by plastic dividers with holes for the next 24 h. Control mice that were not defeated were housed in identical cages with another C57BL6/J mouse without being defeated with the CD1 mouse and were handled throughout the 10-days protocol period.

### Pharmacological Treatment and Behavioral Tests

In experiments to study the therapeutic or prophylactic effects of amphotericin B liposomes, the mice were divided into vehicle, amphotericin B liposome, vehicle + CSDS, and amphotericin B liposome + CSDS groups (10 in each group). To investigate whether stimulation of the innate immune system was required for the therapeutic or prophylactic effect of amphotericin B liposome on CSDS-induced behavioral abnormalities, mice were divided into vehicle, amphotericin B liposome, minocycline, minocycline + amphotericin B liposome, vehicle + CSDS, amphotericin B liposome + CSDS, minocycline + CSDS, and minocycline + amphotericin B liposome + CSDS groups (10 in each group). The amphotericin B liposome was administered intraperitoneally at a dose of 1 mg/kg, following one of our previous studies (Gao et al., 2019), and control mice received saline. Minocycline was injected intraperitoneally 2 days before the amphotericin B liposome injection, followed by further daily injection during behavioral testing to achieve sustained inhibition of the innate immune response. The dose of minocycline (40 mg/kg) was chosen following previous studies (Mika et al., 2009; Lu et al., 2021).

In all experiments, the drug was administered between 08:00 and 10:00 a.m. The social interaction test (SIT), tail suspension test (TST), and forced swimming test (FST), which were performed on the first, second, and third day, respectively, after the stress stimulation or amphotericin B liposome injection, were used to record the changes in abnormal behaviors in mice. The timelines for the different experiments were shown in each figure. All behavioral tests were performed during the light phase.

### Social Interaction Test

The SIT, consisting of two trials of 5 min each, was performed following our previous studies (Gu et al., 2021). In the first trial (target absent), each mouse was allowed to explore a plastic enclosure located in the pre-defined interaction zone. In the second trial (target present), each mouse was returned to the open-field arena, which contained a plastic enclosure in which an unfamiliar CD1 mouse was now setting. The time spent in the interaction zone was recorded using the Ethovision XT (Noldus, United States ) software. The apparatus was cleaned with 70% ethanol after each trial.

### Tail Suspension Test and Forced Swimming Test

The FST and TST were conducted according to our previous studies (Gu et al., 2021). In the FST, the experimental mice were placed in a clear glass cylinder (height 25 cm and diameter 10 cm) filled to 10 cm with water at 25°C ± 1°C for 6 min and defined as immobile when they floated in the water without struggling, making only necessary movements to keep their heads above the water. In the TST, the experimental mice were suspended 50 cm above the floor for 6 min by adhesive tape placed approximately 1 cm from the tip of the tail. Any mouse that climbed its tail was excluded from further analysis. The immobility time (completely motionless) during the last 4 min of forced swimming or tail suspension was recorded by a video (Anhui Zhenghua Biological Instrument Equipment Co., Ltd., Huaibei, China) and manually analyzed using the statistical software in Graphpad Prism 8 (Graphpad Software, Inc., La Jolla, CA, United States).

### Serum Cytokine Measurement

The concentrations of interleukin-1β (IL-1β), IL-6, and tumor necrosis factor-α (TNF-α) in serum were determined using standard kits according to the manufacturer’s protocol (Proteintech, Wuhan, China). The concentrations of IL-1β, IL-6, and TNF-α are expressed as pictograms per milliliter (pg/ml).

### Statistical Analysis

Statistical analyses were performed using Graphpad Prism 8. Differences between the means of these data were evaluated with a two-way analysis of variance (ANOVA), followed by a posthoc Bonferroni test to assess isolated comparisons. *p* values < 0.05 were considered statistically significant. Data are presented as mean ± standard error of the mean (SEM).

## Results

### Effects of Post-Stress Amphotericin B Liposome Injection on CSDS-Induced Behavioral Abnormalities in Mice

**FIGURE 1:**
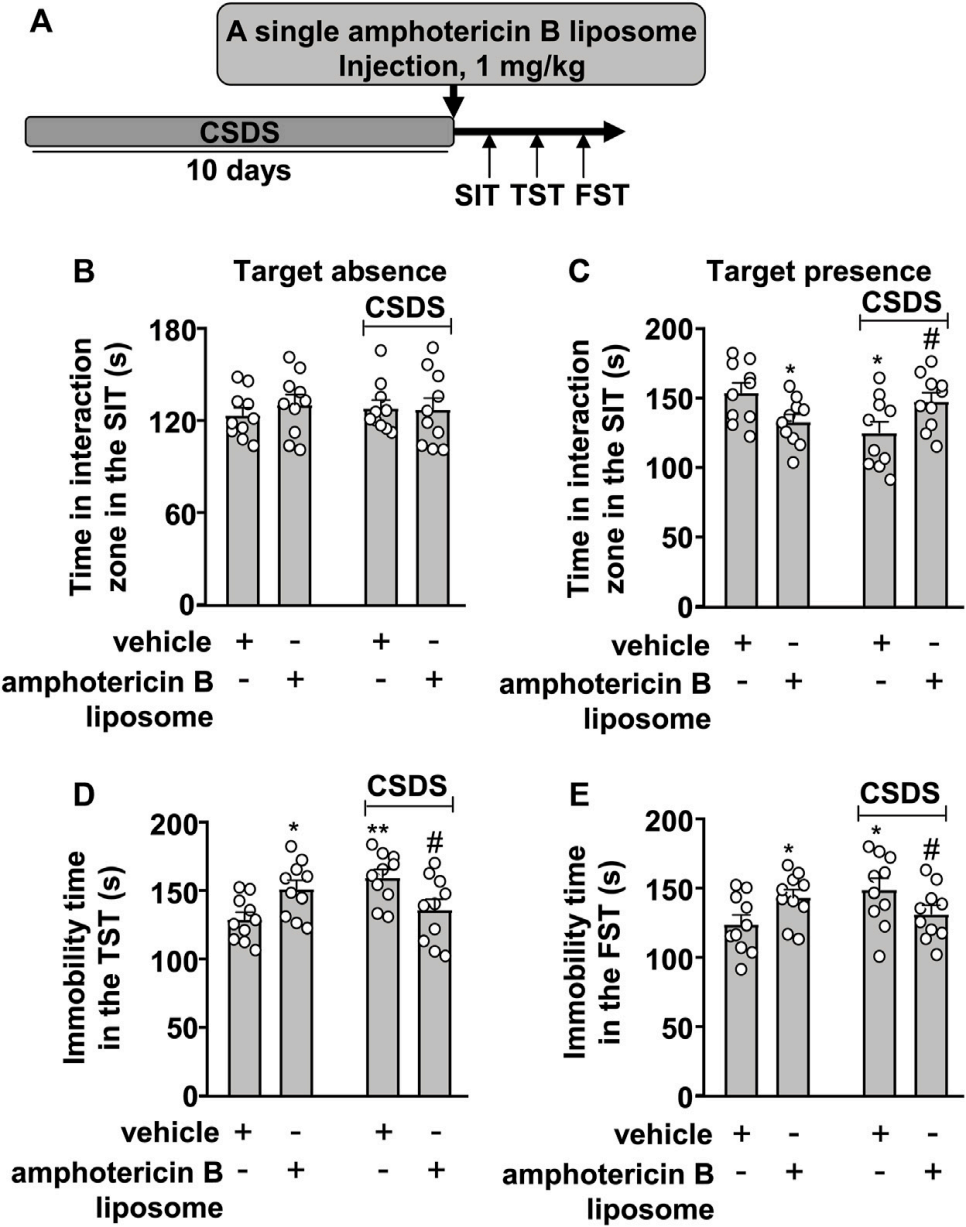
Effect of post-stress amphotericin B liposome administration on CSDS-induced behavioral abnormalities in mice. **(A)** schematic diagram showing the time course of amphotericin B liposome injection after stress and behavioral testing in stress-naïve and CSDS mice. **(B–E)** Quantitative analysis showed that a single injection of amphotericin B liposome (1 mg/kg) after the discontinuation of stress reversed the decreased time spent in the interaction zone in the SIT **(B,C)** and the increased immobility time in the TST **(D)** and FST **(E)** in CSDS mice, and treatment with amphotericin B liposome alone significantly decreased the time spent in the interaction zone in the SIT **(B,C)** and significantly increased the immobility time in the TST **(D)** and FST **(E)** in stress-naïve mice (n = 10, ∗*p* < 0.05 or ∗∗*p* < 0.01 vs. vehicle, #*p* < 0.05 vs. vehicle + CSDS). Data are shown as mean ± SEM.

We first investigated the therapeutic effect of amphotericin B liposomes on CSDS-induced behavioral abnormalities in mice. To this end, mice were administered a single amphotericin B liposome injection at a dose of 1 mg/kg (i.p.), followed by a group of behavioral tests (see Figure 1A for timeline). In the SIT, a two-way ANOVA for the time spent in the interaction zone when the target was absent showed no significant effects for CSDS exposure (F_1.36_ = 0.01, *p* = 0.91), amphotericin B liposome injection (F_1.36_ = 0.23, *p* = 0.64), and the CSDS × amphotericin B liposome interaction (F_1.36_ = 0.41, *p* = 0.53) (Figure 1B), while when the target was present, the two-way ANOVA for the time spent in the interaction zone showed a significant effect for the CSDS × amphotericin B liposome interaction (F_1.36_ = 10.78, *p* < 0.01), but not for CSDS exposure (F_1.36_ = 1.17, *p* = 0.29) and amphotericin B liposome injection (F_1.36_ = 0.01, *p* = 0.91) (Figure 1C). Post hoc analysis revealed that a single injection of amphotericin B liposomes (1 mg/kg) reversed the CSDS-induced reduction in the time spent in the interaction zone in the SIT, whereas in stress-naïve mice, the same amphotericin B liposome injection induced a significant reduction in the time spent in the interaction zone in the SIT (Figure 1C).

For the immobility time in the TST, the two-way ANOVA showed a significant effect for the CSDS × amphotericin B liposome interaction (F_1.36_ = 13.41, *p* < 0.001), but not for CSDS exposure (F_1.36_ = 1.52, *p* = 0.23) and amphotericin B liposome injection (F_1.36_ = 0.01, *p* = 0.91) (Figure 1D), and for the immobility time in the FST, the ANOVA for the immobility time also showed a significant effect for the CSDS × amphotericin B liposome interaction (F_1.36_ = 7.70, *p* < 0.01), but not for CSDS exposure (F_1.36_ = 0.96, *p* = 0.33) and amphotericin B liposome injection (F_1.36_ = 0.02, *p* = 0.88) (Figure 1E). Post hoc analysis revealed that a single injection of amphotericin B liposomes (1 mg/kg) reversed the CSDS-induced increase in the immobility time in the TST (Figure 1D) and FST (Figure 1E), whereas in stress-naïve mice, the same amphotericin B liposome injection resulted in significant increases in the immobility time in the TST (Figure 1D) and FST (Figure 1E).

### Effects of Pre-Stress Amphotericin B Liposome Injection on CSDS-Induced Behavioral Abnormalities in Mice

**FIGURE 2:**
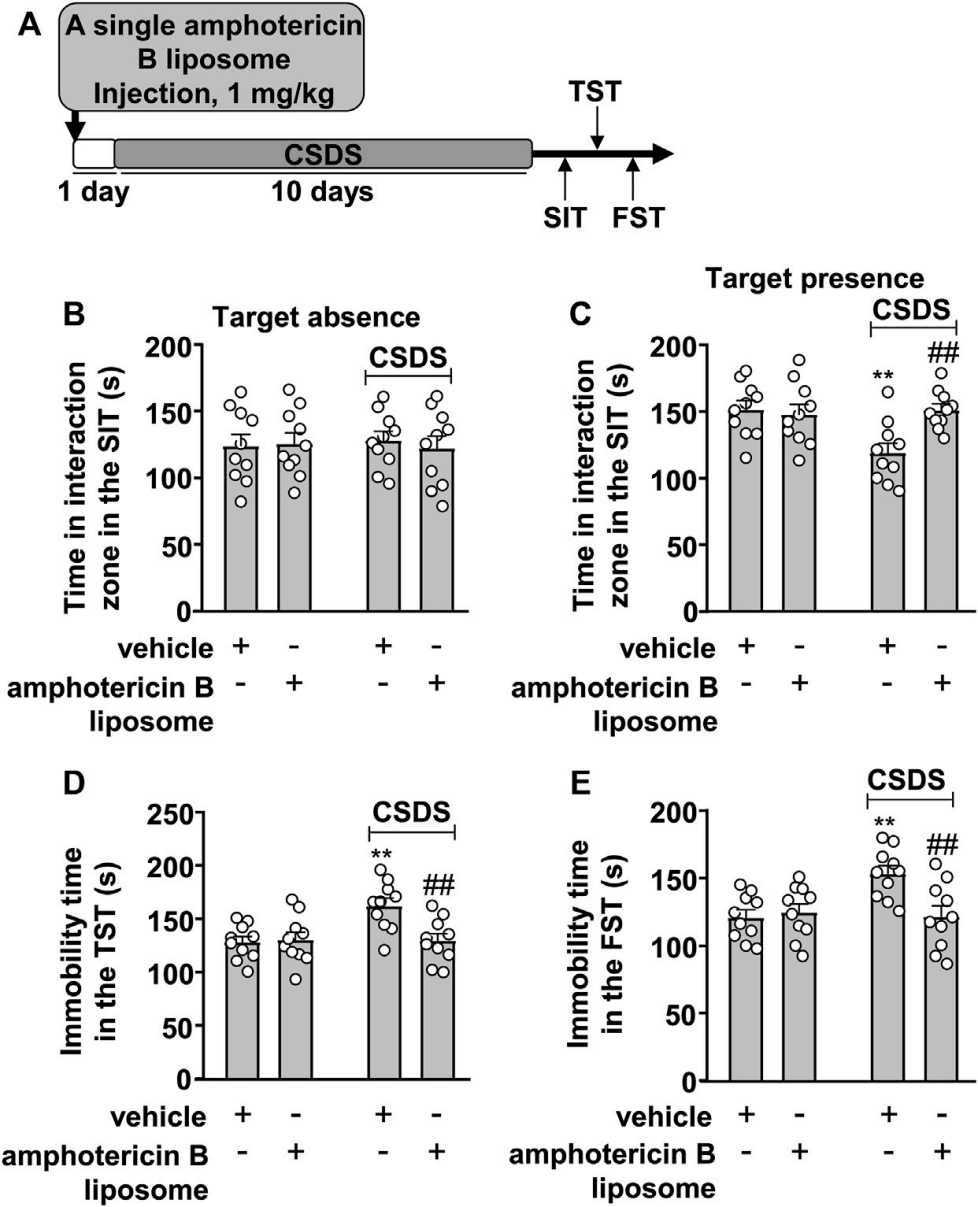
Effect of pre-stress amphotericin B liposome administration on CSDS-induced behavioral abnormalities in mice. **(A)** schematic diagram showing the time course of amphotericin B liposome injection before stress exposure and behavioral testing in stress-naïve and CSDS mice. **(B–E)** Quantitative analysis showed that a single injection of amphotericin B liposome (1 mg/kg) 1 day before stress exposure prevented the CSDS-induced decrease in the time spent in the interaction zone in the SIT **(B,C)** and the CSDS-induced increases in the immobility time in the TST **(D)** and FST **(E)** in mice (n = 10, ∗∗*p* < 0.01 vs. vehicle, ##*p* < 0.01 vs. vehicle + CSDS). Data are shown as mean ± SEM.

The prophylactic effect of amphotericin B liposomes on CSDS-induced behavioral abnormalities was also investigated (Figure 2A). In the SIT, a two-way ANOVA for the time spent in the interaction zone when the target was absent showed no significant effects for CSDS exposure (F_1.36_ = 0.001, *p* = 0.97), amphotericin B liposome injection (F_1.36_ = 0.06, *p* = 0.81), and the CSDS × amphotericin B liposome interaction (F_1.36_ = 0.21, *p* = 0.65) (Figure 2B), while when the target was present, the two-way ANOVA for the time spent in the interaction zone showed significant effects for CSDS exposure (F_1.36_ = 4.93, *p* < 0.05), amphotericin B liposome injection (F_1.36_ = 4.77, *p* < 0.05), and the CSDS × amphotericin B liposome interaction (F_1.36_ = 7.46, *p* < 0.01) (Figure 2C). Post hoc analysis revealed that a single pre-injection of amphotericin B liposomes (1 mg/kg) 1 day before stress exposure prevented the CSDS-induced reduction in the time spent in the interaction zone in the SIT (Figures 2B,C).

For the TST, the two-way ANOVA for the immobility time showed significant effects for CSDS exposure (F_1.36_ = 6.52, *p* < 0.05), amphotericin B liposome injection (F_1.36_ = 5.39, *p* < 0.05), and the CSDS × amphotericin B liposome interaction (F_1.36_ = 7.17, *p* < 0.05) (Figure 2D), and for the immobility time in the FST, the ANOVA also showed significant effects for CSDS exposure (F_1.36_ = 5.33, *p* < 0.05), amphotericin B liposome injection (F_1.36_ = 4.85, *p* < 0.05), and the CSDS × amphotericin B liposome interaction (F_1.36_ = 7.76, *p* < 0.01) (Figure 2E). Post hoc analysis revealed that a single pre-injection of amphotericin B liposomes (1 mg/kg) 1 day before stress exposure prevented the CSDS-induced increases in the immobility time in the TST (Figure 2D) and FST (Figure 2E).

### Innate Immune Inhibition Abolishes the Reversal Effect of Amphotericin B Liposome on CSDS-Induced Behavioral Abnormalities in Mice

**FIGURE 3:**
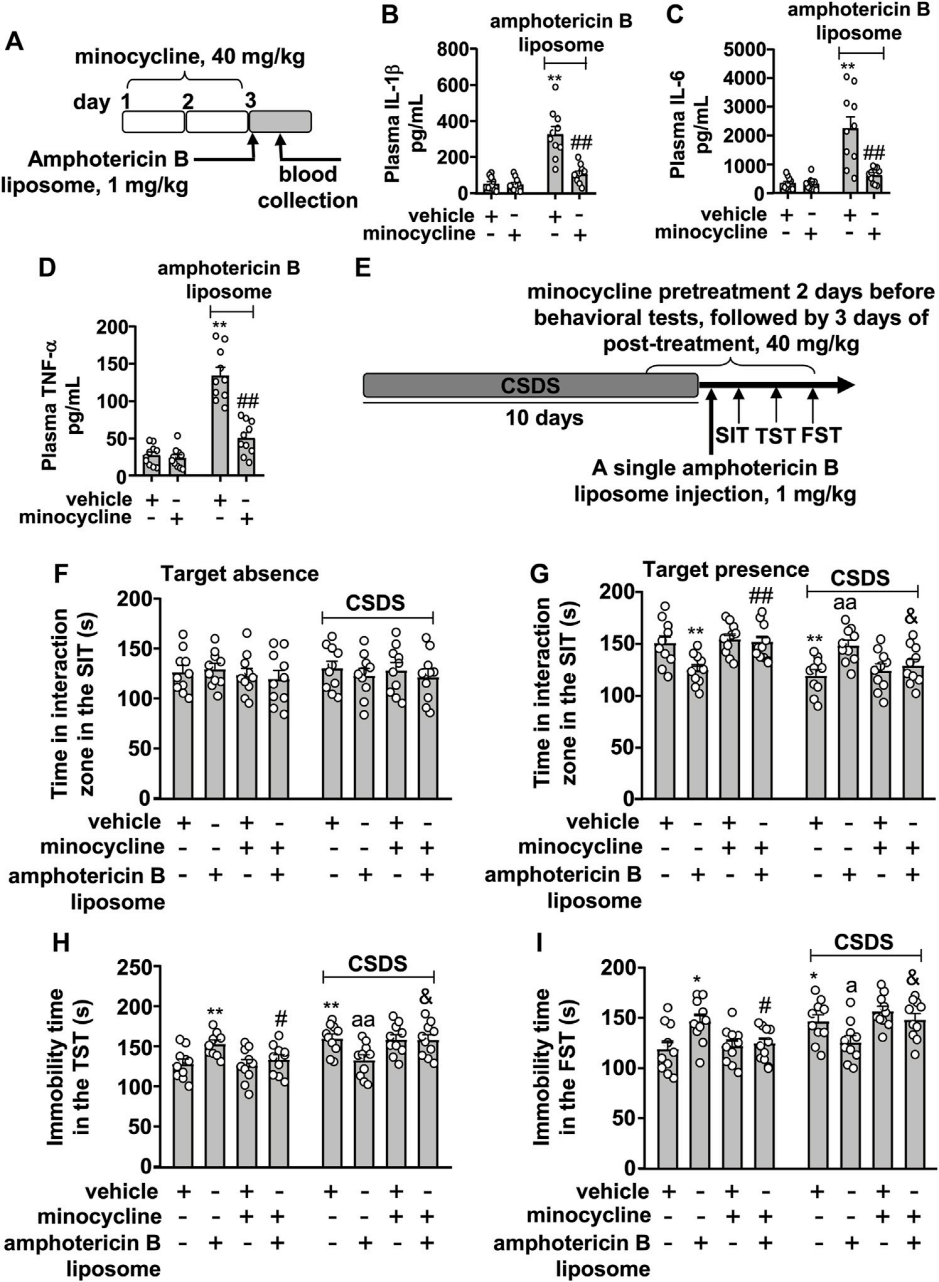
Effect of minocycline pretreatment on amphotericin B liposome-induced reversal of CSDS-induced behavioral abnormalities in mice. **(A)** schematic diagram shows the experimental setup for minocycline pretreatment and amphotericin B liposome injection in stress-naïve mice. **(B–D)** Quantitative analysis shows the preventive effect of minocycline pretreatment (40 mg/kg) on the increases in blood IL-1 β **(B)**, IL-6 **(C)**, and TNF-α **(D)** levels induced by injection of amphotericin B liposomes (1 mg/kg) in stress-naïve mice (n = 10, ∗∗*p* < 0.01 vs. vehicle; ##*p* < 0.01 vs. vehicle + amphotericin B liposome). **(E)** A schematic diagram shows the time course of CSDS, SIT, TST, FST, minocycline pretreatment, and/or post-stress amphotericin B liposome injection. **(F–I)** Quantitative analysis showed that minocycline pretreatment (40 mg/kg) abolished the single amphotericin B liposome injection (1 mg/kg)-induced reversal of CSDS-induced decrease in the time spent in the interaction zone in the SIT **(F,G)** and CSDS-induced increases in the immobility time in TST **(H)** and FST **(I)** (n = 10, ∗*p* < 0.05 or ∗∗*p* < 0.01 vs. vehicle; ap < 0.05 or aap < 0.01 vs. vehicle + CSDS; &*p* < 0.05 vs. amphotericin B liposome + CSDS), and minocycline pretreatment (40 mg/kg) prevented the single amphotericin B liposome injection (1 mg/kg)-induced decrease in the time spent in the interaction zone in the SIT **(G)** and the amphotericin B liposome-induced increases in the immobility time in TST **(H)** and FST **(I)** in stress-naïve mice (n = 10, ∗*p* < 0.05 or ∗∗*p* < 0.01 vs. vehicle; #*p* < 0.05 or ##*p* < 0.01 vs. amphotericin B liposome). Data are shown as mean ± SEM.

Next, we investigated whether stimulation of the innate immune system mediates the therapeutic effect of amphotericin B liposomes on CSDS-induced behavioral abnormalities. For this purpose, we collected a blood sample to verify the inhibitory effect of minocycline on the innate immune response (Figure 3A). The results showed that minocycline pretreatment (40 mg/kg) counteracted the increases in plasma levels of IL-1β (Figure 3B; two-way ANVOA: significant effects for amphotericin B liposome injection (F_1.36_ = 48.91, *p* < 0.001), minocycline pretreatment (F_1.36_ = 22.43, *p* < 0.001), and the amphotericin B liposome × minocycline interaction (F_1.36_ = 20.86, *p* < 0.001)), IL-6 (Figure 3C; two-way ANVOA: significant effect for amphotericin B liposome injection (F_1.36_ = 28.28, *p* < 0.001), minocycline pretreatment (F_1.36_ = 15.69, *p* < 0.001), and the amphotericin B liposome × minocycline interaction (F_1.36_ = 15.29, *p* < 0.001)), and TNF-α (Figure 3D; two-way ANVOA: significant effect for amphotericin B liposome injection (F_1.36_ = 87.75, *p* < 0.001), minocycline pretreatment (F_1.36_ = 37.64, *p* < 0.001), and the amphotericin B liposome × minocycline interaction (F_1.36_ = 31.49, *p* < 0.001)) induced by amphotericin B liposome (1 mg/kg), suggesting that the minocycline used here is sufficient to suppress activation of the innate immune response triggered by amphotericin B liposomes.

Based on the above results, we investigated the effect of minocycline pretreatment on the reversal of behavioral abnormalities in CSDS mice (Figure 3E). As shown in Figures 3A,F,G two-way ANOVA for the time spent in the interaction zone when the target was absent in the SIT showed no significant effects for CSDS exposure (F_1.72_ = 0.05, *p* = 0.83), amphotericin B liposome/minocycline treatment (F_3.72_ = 0.43, *p* = 0.73), and the CSDS × amphotericin B liposome/minocycline interaction (F_3.72_ = 0.26, *p* = 0.86), while when the target was present, the two-way ANOVA for the time spent in interaction zone showed significant effects for CSDS exposure (F_1.72_ = 14.72, *p* < 0.001) and the CSDS × amphotericin B liposome/minocycline interaction (F_3.72_ = 10.78, *p* < 0.001), but not for amphotericin B liposome/minocycline treatment (F_3.72_ = 0.40, *p* = 0.75). Post hoc analysis revealed that minocycline pretreatment (40 mg/kg) almost completely abolished the increase in the time spent in the interaction zone in the SIT in CSDS mice induced by post-stress amphotericin B liposome injection (Figures 3F,G) and that minocycline pretreatment prevented the decrease in the time spent in the interaction zone in the SIT induced by amphotericin B liposome injection (1 mg/kg) in stress-naïve mice (Figure 3G).

For the immobility time in the TST, the two-way ANOVA showed significant effects for CSDS exposure (F_1.72_ = 15.50, *p* < 0.001) and the CSDS × amphotericin B liposome/minocycline interaction (F_3.72_ = 8.60, *p* < 0.001), but not for amphotericin B liposome/minocycline treatment (F_3.72_ = 0.12, *p* = 0.95) (Figure 3H), and for the immobility time in the FST, the ANOVA showed significant effects for CSDS exposure (F_1.72_ = 13.47, *p* < 0.001) and the stress × amphotericin B liposome/minocycline interaction (F_3.72_ = 8.10, *p* < 0.001), but not for amphotericin B liposome/minocycline treatment (F_3.72_ = 0.38, *p* = 0.77) (Figure 3I). Post hoc analysis revealed that minocycline pretreatment (40 mg/kg) was able to abolish the reduction in immobility time in the TST (Figure 3H) and FST (Figure 3I) induced by post-stress amphotericin B liposome injection (1 mg/kg) in CSDS mice, and meanwhile, minocycline pretreatment prevented the increases in the immobility time in the TST (Figure 3H) and FST (Figure 3I) induced by amphotericin B liposome injection (1 mg/kg) in stress-naïve mice.

### Innate Immune Inhibition Abolishes the Prophylactic Effect of Amphotericin B Liposome on CSDS-Induced Behavioral Abnormalities in Mice

**FIGURE 4:**
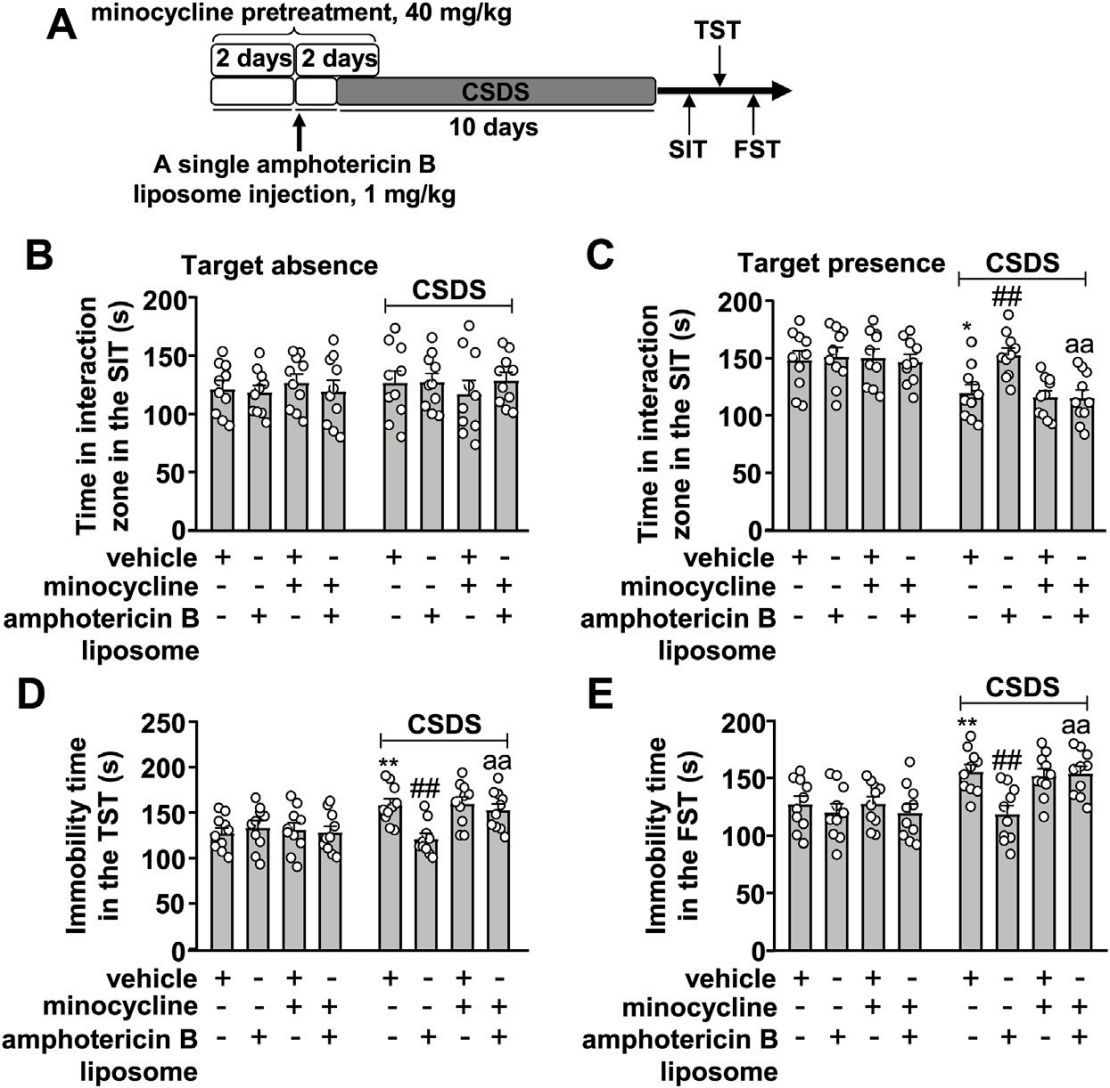
Effect of minocycline pretreatment on amphotericin B liposome preconditioning-induced prevention of CSDS-induced behavioral abnormalities in mice. **(A)** schematic diagram showing the experimental setup for minocycline pretreatment and/or amphotericin B liposome injection in stress-naïve and CSDS mice. **(B–E)** Quantitative analysis showed that minocycline pretreatment (40 mg/kg) abolished the single amphotericin B liposome (1 mg/kg)-induced prevention of CSDS-induced decrease in the time spent in the interaction zone in the SIT **(B,C)** and CSDS-induced increases in the immobility time in TST **(D)** and FST **(E)** (n = 10, ∗*p* < 0.05 or ∗∗*p* < 0.01 vs. vehicle; ##*p* < 0.01 vs. vehicle + CSDS; aap < 0.01 vs. amphotericin B liposome + CSDS). Data are shown as mean ± SEM.

On the basis of the successful inhibition of the innate immune response elicited by amphotericin B liposomes by minocycline in Figures 3A‒D, we investigated whether inhibition of the innate immune system could influence the prophylactic effect of amphotericin B liposomes on CSDS-induced behavioral abnormalities (Figure 4A). As shown in Figures 4A–C two-way ANOVA for the time spent in the interaction zone when the target was absent in the SIT showed no significant effects for CSDS exposure (F_1.72_ = 0.35, *p* = 0.55), amphotericin B liposome/minocycline pretreatment (F_3.72_ = 0.03, *p* = 0.99), and the CSDS × amphotericin B liposome/minocycline interaction (F_3.72_ = 0.58, *p* = 0.63), while when the target was present, the two-way ANOVA for the time spent in interaction zone showed significant effects for CSDS exposure (F_1.72_ = 22.55, *p* < 0.001), amphotericin B liposome/minocycline treatment (F_3.72_ = 3.91, *p* < 0.05), and the CSDS × amphotericin B liposome/minocycline interaction (F_3.72_ = 2.81, *p* < 0.05). Post hoc analysis revealed that minocycline pretreatment (40 mg/kg) abolished the increase in the time spent in the interaction zone in the SIT in CSDS mice induced by pre-stress amphotericin B liposome injection (1 mg/kg; Figures 4B,C).

For the immobility time in the TST, the two-way ANOVA showed significant effects for CSDS exposure (F_1.72_ = 14.03, *p* < 0.001), amphotericin B liposome/minocycline treatment (F_3.72_ = 2.80, *p* < 0.05), and the CSDS × amphotericin B liposome/minocycline interaction (F_3.72_ = 4.43, *p* < 0.01) (Figure 4D), and for the immobility time in the FST, the ANOVA showed significant effects for CSDS exposure (F_1.72_ = 19.86, *p* < 0.001), amphotericin B liposome/minocycline treatment (F_3.72_ = 4.57, *p* < 0.01), and the CSDS × amphotericin B liposome/minocycline interaction (F_3.72_ = 2.78, *p* < 0.05) (Figure 4E). Post hoc analysis revealed that minocycline pretreatment (40 mg/kg) abolished the reduction in the immobility time in the TST (Figure 4D) and FST (Figure 4E) induced by pre-stress amphotericin B liposome injection (1 mg/kg) in CSDS mice.

## Discussion

One of the major findings of the present study was that amphotericin B liposome, a compound approved by the Food and Drug Administration for the treatment of fungal infections, reversed CSDS-induced behavioral abnormalities in mice by mobilizing the innate immune response, which is consistent with some previous findings that stimulation of the innate immune system by a single LPS, M-CSF, or GM-CSF injection reverses chronic stress-induced behavioral abnormalities in mice (Kreisel et al., 2014; Tong et al., 2017; Cai et al., 2020; Ye et al., 2020). CSDS exposure is a commonly used psychological stressor that causes depressive signs in rodents. During this exposure, the SIT is typically used to assess changes in animal behaviors reflecting their social interaction ability (Toth and Neumann, 2013). The TST and the FST, which were used here, are also common behavioral tests that indicate the antidepressant efficacy of antidepressant substances (Porsolt et al., 1977; Steru et al., 1985) and may indicate learned helplessness, the latter part of which could be a symptom of depression. In light of this, our present results may indicate that stimulation of the innate immune system through immune preconditioning could be a potential strategy to prevent mental disorders including depression.

In research on mental disorders, the neuroiniflammation hypothesis has attracted much attention, so suppression of neuroinflammation is considered a possible strategy to treat mental disorders (Dey and Hankey Giblin, 2018; Li et al., 2022; Tateishi et al., 2022; Tayab et al., 2022). However, in recent years, due to the heterogeneity of factors underlying the pathogenesis of mental disorders, it has been suggested that mental disorders should be treated by a personalized medical approach (Yirmiya et al., 2015). In patients whose central innate immune response is hypofunctional, a stimulant of the innate immune system should be administered to alleviate the symptoms of mental disorders (Yirmiya et al., 2015). Indeed, a number of previously published studies have supported this hypothesis: 1) depressed patients have lower activation of microglia and lower levels of pro-inflammatory markers in serum and brain (Whooley et al., 2007; Almeida et al., 2009; Dowlati et al., 2010; Lehto et al., 2010), which could indicate a hypofunctional state of the innate immune system, 2) some anti-inflammatory drugs such as NSAIDs and COX-1 inhibitors may induce or exacerbate depressive symptoms (Browning, 1996; Choi et al., 2009), 3) some pro-inflammatory mediators such as TNF-α and interferon-γ (IFN-γ), may ameliorate but not exacerbate depressive signs in animals (Warner-Schmidt et al., 2011). Our results on the therapeutic effect of amphotericin B liposomes on CSDS-induced behavioral abnormalities, as evidenced by the behavioral changes in the SIT, TST, and FST, provide further evidence for the importance of innate immune system stimulation in ameliorating chronic stress-induced behavioral abnormalities and suggest that amphotericin B liposomes could be developed as a novel drug for the treatment of mental disorders.

Another important finding of the present study was that a single injection of amphotericin B liposomes 1 day before stress exposure had a preventive effect on CSDS-induced behavioral abnormalities in mice. This is likely mediated by stimulation of the innate immune system and is consistent with our previous findings that pre-stimulation of the innate immune system by a single low-dose LPS or M-CSF injection 1 day before stress exposure prevents the onset of behavioral abnormalities in CSDS mice (Gu et al., 2021; Ji et al., 2021) and showed that amphotericin B liposomes could be developed as a novel drug to prevent the onset of mental disorders. This could be of great importance to reduce the morbidity of mental disorders from the root and to reduce the social and economic burden caused by mental disorders. Preconditioning of the innate immune system is an old concept, but its prophylactic effect on central nervous system disorders, including cerebral ischemia and brain trauma, remains impressive. In the present study, we found that an inhibitor of the innate immune response, minocycline, abolished the prophylactic effect of amphotericin B liposomes on CSDS-induced behavioral abnormalities, suggesting that the innate immune response is required to enable the preventive effect of amphotericin B liposomes on CSDS-induced behavioral abnormalities.

The next question to answer is how the administration of amphotericin B liposomes reverses or prevents the behavioral abnormalities triggered by chronic stress. We first focused on microglia because the contributions of microglia to the therapeutic and prophylactic effects of innate immune system stimulants on central nervous system disorders have been described in detail in previous studies (Mirrione et al., 2010; Matsuda et al., 2015; Gu et al., 2021). For example, it has been reported that the decline of microglia in the dentate gyrus of the hippocampus under chronic stress exposure mediates the pathogenesis of behavioral abnormalities and that reversal of this decline by innate immune system stimulants ameliorates chronic stress-induced behavioral abnormalities (Kreisel et al., 2014; Tong et al., 2017; Cai et al., 2020). Our previous studies had also reported that depletion of microglia in the hippocampus abolished the therapeutic effect of a single low dose of LPS preconditioning on chronic stress-induced behavioral abnormalities in mice (Cai et al., 2020). In animal models of traumatic brain injury or epilepsy, activation of microglia has been reported to mediate the neuroprotective effect of LPS preconditioning on neuronal damage (Mirrione et al., 2010; Longhi et al., 2011; Matsuda et al., 2015). Therefore, it is reasonable to hypothesize that the amphotericin B liposome has therapeutic and prophylactic effects on CSDS-induced behavioral abnormalities, probably through activation of microglia in the brain. However, in addition to microglia, the role of other types of innate immune cells, such as macrophages and T cells, in the therapeutic and prophylactic effects of amphotericin B liposomes on chronic stress-induced behavioral abnormalities should also be considered, because it has been shown that preconditioning macrophages with a synthetic malaria pigment reduces the production of proinflammatory cytokines (Taramelli et al., 2000) and suppression of T-cell function can abrogate the behavioral improvement effect of a heat-killed preparation of Mycobacterium vaccae in a psychiatric model induced by stress from social defeat (Reber et al., 2016). Therefore, amphotericin B liposome may have therapeutic and prophylactic effects on CSDS-induced behavioral disorders by stimulating macrophages or T cells.

Regardless of which cell types mediate the therapeutic effect of amphotericin B liposomes on CSDS-induced behavioral abnormalities, an important question that should be answered is how the innate immune response mobilized by amphotericin B liposome causes a reversal effect on CSDS-induced behavioral abnormalities. According to the hypothesis of microglia loss in depression (Kreisel et al., 2014; Tong et al., 2017; Cai et al., 2020), we hypothesize that the behavior-improving effects of amphotericin B liposome are mediated by its reversal effect on the CSDS-induced decline of microglia in the hippocampus. After amphotericin B liposome injection, the restored functional microglia might interact with other cells in the brain, such as neurons and astrocytes, or release endogenous molecules that modify brain function, such as adenosine triphosphate, nitric oxide, and glutamate, thus promoting neurotransmission or neurogenesis and leading to the improvement of depressive symptoms. These hypotheses should be investigated in future studies.

How pre-injection of amphotericin B liposomes prevents the behavioral abnormalities triggered by chronic stress remains to be elucidated. Activation of the innate immune system is known to be mediated by different types of immune cells in the body, such as microglia, monocytes, macrophages, and peripheral T cells. The contributions of these cells to the protective effects of innate immune stimulation in various types of pathological models have been demonstrated in previous studies. For example, activation of microglia was confirmed to be essential for the neuroprotective effect of a low-dose LPS pretreatment in models of epilepsy (Hosseinzadeh et al., 2019; Ohgomori and Jinno, 2020) and traumatic brain injury (Longhi et al., 2011; Chen et al., 2014). LPS-preconditioned monocytes can mobilize from the spleen and reach the brain, where they ameliorate brain damage induced by transient middle cerebral artery occlusion by suppressing the progression of neuroinflammation (Garcia-Bonilla et al., 2018). Suppression of the T-cell function can abolish the protective effect of a heat-killed preparation of Mycobacterium vaccae in a psychiatric model induced by social defeat stress (Reber et al., 2016). In addition, stimulation of the innate immune response by Toll-like receptor agonists was found to enhance tumor destruction by adoptively transferred CD8 (+) T cells (Nelson et al., 2016). The exact cellular basis for the prophylactic effect of amphotericin B liposomes should be investigated in future studies.

It is well known that the pathogenesis of mental disorders could be explained by a variety of hypotheses, such as the neuroinflammation hypothesis (Dey and Hankey Giblin, 2018; Li et al., 2022), the cyclic adenosine monophosphate response element binding protein (CREB)-brain-derived neurotrophic factor (BDNF) signaling impairment hypothesis (Koch et al., 2009; Tan et al., 2022), and the neurotransmission dysfunction hypothesis (Wang et al., 2021; Yan and Rein, 2022). The neuroprotective effect of LPS preconditioning in cerebral ischemia and traumatic brain injury has been reported to be associated with its preventive effect on the pathological progression of neuroinflammation through mechanisms such as nuclear factor-κB (NF-κB) inhibition and interferon regulatory factor (IRF) activation (Stevens et al., 2011; Vartanian et al., 2011). In our previous studies, we reported that a single low-dose LPS injection prevented the development of behavioral abnormalities in mice by converting the neuroinflammatory responses into an anti-inflammatory phenotype (Gu et al., 2021). Therefore, it is reasonable to speculate that the prophylactic effect of amphotericin B liposome on CSDS-induced behavioral abnormalities may be mediated by inhibition of neuroinflammation. We also noticed that vaginal LPS preconditioning can rescue the amniotic LPS-induced apoptosis in fetal brains through a marked increase in phosphorylation levels of CREB at Ser133 (Dong et al., 2022), and displacement of inhibitory GABAergic synapses by LPS-mobilized microglia can increase synchronous firing of cortical neurons in the γ-frequency band and reduce apoptosis of cortical neurons after injury, with marked increases in phospho-CREB and BDNF levels (Chen et al., 2014). Moreover, LPS preconditioning in early life was found to ameliorate cecal ligation and perforation (CLP)-induced memory impairment in adult rats and to increase the expression of BDNF in the brain (Florentino et al., 2020). Thus, the prophylactic effect of amphotericin B liposome on chronic stress-induced behavioral abnormalities in mice could also be mediated by its preventive effect on the disrupted CREB-BDNF signaling pathway in the brain. Because CSDS exposure and disruption of CREB-BDNF signaling are closely related to the impairment of neurogenesis in the brain (Maitra et al., 2020; Jiang et al., 2021; Gao et al., 2022) and stimulation of the innate immune system can promote neurogenesis (Reshef et al., 2017; Al-Onaizi et al., 2020; Seong et al., 2022), the prophylactic effect of amphotericin B liposomes on chronic stress-induced behavioral abnormalities might also be mediated by enhancing neurogenesis through improving CREB-BDNF signaling function. These uncertainties should be explored in future studies.

## Conclusion

Our results demonstrate that a single injection of amphotericin B liposomes can have both therapeutic and prophylactic effects on CSDS-induced behavioral abnormalities in mice by stimulating the innate immune system. These results not only demonstrate the importance of innate immune response in the prevention and treatment of mental disorders but also show that amphotericin B liposomes may be a candidate for the prevention or treatment of mental disorders. Future studies should further clarify the cellular and molecular basis for the therapeutic and prophylactic effects of amphotericin B liposomes on CSDS-induced behavioral abnormalities and mental disorders including depression.

## Data Availability

The raw data supporting the conclusions of this article will be made available by the authors, without undue reservation.
